# Identification and Functional Characterization of the Soybean *GmaPPO12* Promoter Conferring *Phytophthora sojae* Induced Expression

**DOI:** 10.1371/journal.pone.0067670

**Published:** 2013-06-28

**Authors:** Chunyue Chai, Yanling Lin, Danyu Shen, Yuren Wu, Hongjuan Li, Daolong Dou

**Affiliations:** 1 Department of Plant Pathology, Nanjing Agricultural University, Nanjing, China; 2 College of Life Science and Technology, Nanyang Normal University, Nanyang, China; Instituto Valenciano de Investigaciones Agrarias, Spain

## Abstract

Identification of pathogen-inducible promoters largely lags behind cloning of the genes for disease resistance. Here, we cloned the soybean *GmaPPO12* gene and found that it was rapidly and strongly induced by *Phytophthora* *sojae* infection. Computational analysis revealed that its promoter contained many known *cis*-elements, including several defense related transcriptional factor-binding boxes. We showed that the promoter could mediate induction of GUS expression upon infection in both transient expression assays in *Nicotiana* *benthamiana* and stable transgenic soybean hairy roots. Importantly, we demonstrated that pathogen-induced expression of the *GmaPPO12* promoter was higher than that of the soybean *GmaPR1a* promoter. A progressive 5’ and 3’ deletion analysis revealed two fragments that were essential for promoter activity. Thus, the cloned promoter could be used in transgenic plants to enhance resistance to *phytophthora* pathogens, and the identified fragment could serve as a candidate to produce synthetic pathogen-induced promoters.

## Introduction

Soybean (*Glycine max* [L.] Merr.) is a legume crop of great economic and agricultural importance across the world. Soybean yields are significantly reduced due to *Phytophthora* root and stem rot caused by *Phytophthora* *sojae* . The disease leads to 1-2 billon dollars in damage globally every year [1]. *P* *. sojae* is an oomycete, which is most closely related to algae such as kelp and diatoms, distinct from fungi and a member of the stramenopiles [1–3]. Oomycetes contain many other devastating plant pathogens, such as *Phytophthorainfestans* responsible for potato and tomato late blight [4], and *Plasmopara* *viticola* and *Pseudoperonospora* *cubensis* causing downy mildew on grape and cucumber, respectively [5]. Breeding for disease resistance is the main strategy for combating oomycete diseases. In soybean, 15 *Rps* (resistance to *P* *. sojae* ) genes that have been mapped to nine genomic loci are widely used for breeding [6]. Unfortunately, their effectiveness is often transient because pathogens can overcome disease resistance genes after they are widely and/or continually implicated [7]. Genetic engineering has the potential to provide a complement to some of the weaknesses by expressing antimicrobial compounds or components of known defense signaling pathways that confer durable and broad-spectrum resistance [8].

Several distinct strategies have been used in transgenic plants to enhance oomycete disease resistance, such as modifying the resistance signaling pathway by altering the expression of endogenous components, expressing antimicrobial peptides or pathogenesis-related proteins and even pyramiding the cloned *R* genes [9,10]. A bean gene encoding a polygalacturonase-inhibiting protein (PGIP), which is antagonistic to pathogen polygalacturonase, can protect transgenic tobacco against oomycetes ( *P* *. parasitica* and *Peronosporahyoscyami* ) [11]. Transgenic tobacco overexpressing the polyamine oxidase (PAO) gene, catalyzing the oxidative catabolism of spermidine and spermine to generate hydrogen peroxide, shows pre-induced disease tolerance against *Phytophthora* *parasitica* through activation of the plant systemic acquired resistance (SAR) pathway [12]. Functional stacking of three broad-spectrum potato *R* genes, *Rpi-sto1*, *Rpi-vnt1.1*, and *Rpi-blb3*, in susceptible potato leads to a sum of the resistant spectra from the three individual *Rpi* genes [13]. Despite all efforts, however, the use of transgenic crops that are resistant to oomycete diseases is generally not as successful as conventional plant breeding [8]. This unexpected failure is often due to overexpression of the genes used for disease resistance, leading to a fitness penalty in normal plant growth and development. One possible way to solve the problem is to restrict expression of the transgenic target gene to only when it is needed at the infection sites using pathogen-induced promoters. However, discovery of these promoters largely lags behind identification of the genes for disease resistance [14].

Recently, several pathogen-induced promoters were identified in *Arabidopsis* , tobacco, and rice [14,15]. One of the best studied pathogen-induced promoters is from the tobacco *hsr203J* gene whose activation is rapidly induced by incompatible interaction and selectively expressed in response to the hypersensitive response (HR)-inducible bacteria [16]. Transgenic tobacco plants harboring a fusion between the *hsr203J* promoter and an elicitor (cryptogein) encoding gene from *P* *. cryptogea* exhibit HR resembling cell death when infected with several fungal and oomycete pathogens, thus displaying enhanced broad-spectrum disease resistance [17]. Similar results were also observed when the *hsr203J* promoter fused with *popA* was expressed in tobacco, although some transgenic lines showed runaway cell death [18]. In contrast, this promoter appears to be very weakly activated following pathogen inoculation in transgenic pears [19]. Thus, the suitability of the *hsr203J* promoter in different plant-microbe interaction systems remains unclear and the useful pathogen-inducible promoters are still insufficient.

Pathogen-inducible promoters usually possess many conserved *cis*-regulatory elements that are potential binding sites for pathogen-responsive transcription factors [14,20]. Since each element may respond to one or more specific defense signaling pathways, the expression profile of the targeted gene is usually complex and obtaining a promoter that is not only highly specific to but also rapidly and strongly induced by pathogen infections is challenging. One strategy to overcome this complexity is to produce synthetic promoters by stacking or combining only well-characterized individual elements [15,21,22]. Two synthetic promoters, for example, were shown to combine good inducibility with low background in transgenic *Arabidopsis* [22]. Although testing these synthetic promoters in crop plants for disease resistance is still necessary, use of the synthetic promoters for pathogen phytosensing could potentially monitor plant disease outbreaks in agricultural fields [23]. Considering that rapid *in vivo* analysis of synthetic pathogen-inducible promoters is available [15,24], identifying functional *cis*-elements in crop plants is necessary.

Here, a *P* *. sojae* -induced promoter from the soybean *GmaPPO12* gene was identified and characterized using stable transgenic soybean hairy roots and transient expression assays in *N.* benthamiana. Our results demonstrated that the gene was an immediate-early *P* *. sojae* -induced gene. Deletion mutant analysis revealed that a 113-bp fragment in the *GmaPPO12* promoter is important for the activities. To our understanding, this is the first report of a pathogen-induced promoter in soybeans.

## Results

### Soybean *GmaPPO12* is highly induced by *P* *. sojae*

**Figure 1:**
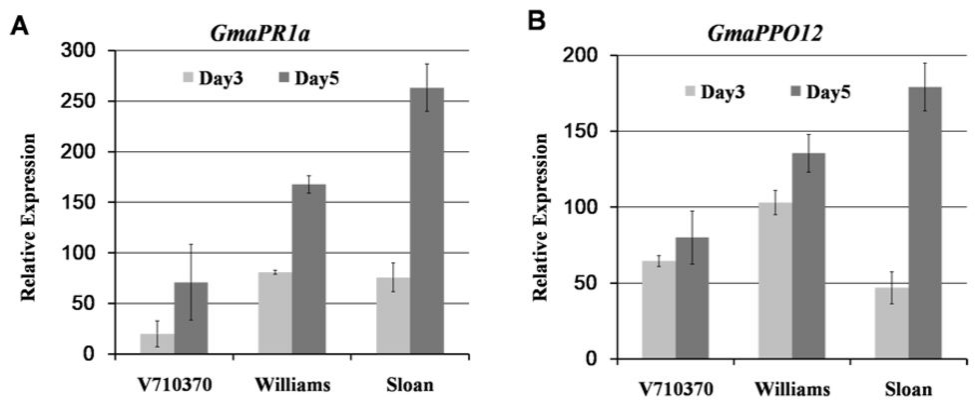
The microarray data of relative expression levels of *GmaPPO12* (A) and *GmaPR1a* (B). Relative expression levels of *GmaPPO12* (A) and *GmaPR1a* (B) in three different soybean cultivars (V710370, Williams and Sloan) were collected from the published data and calculated for relative fold change compared to these in mock samples. The error bar indicates the fold change in three independent experiments.

Recently, a large-scale microarray experiment involving three soybean cultivars and 72 biological replicates was carried out to analyze the response of soybean plants to infection by the pathogen *P* *. sojae* . The overall results showed that almost the entire plant genome undergoes significant transcriptional modulation in response to infection and genetic variation [25]. Here, we took advantage of the available microarray data to screen genes that were highly induced by infection with *P* *. sojae* . A gene annotated as Gma.7559.1.S1_s_at was one of the most strongly induced genes. This gene was upregulated 65-fold at 3 dpi (days post inoculation) and 80-fold at 5 dpi in the resistant cultivar V71-370, upregulated 103-fold at 3 dpi and 135-fold at 5 dpi in the moderately resistant cultivar Williams, and upregulated 47-fold at 3 dpi and 179-fold at 5 dpi in the susceptible cultivar Sloan. The expression levels of Gma.7559.1.S1_s_at at 3 dpi were higher than that of the *PR1a* gene in the resistant and moderately resistant cultivars and weaker than the *PR1a* gene in the susceptible cultivar (Figure 1).

Phylogenetic and functional domain analysis showed that this gene belongs to the soybean polyphenol oxidase (PPO) gene family (Figure S1). In soybean, 11 *PPO* genes have been identified and designated as *GmaPPO1-11* [26]. Gma.7559.1.S1_s_at was not identified as one of these genes, and we found that it was an annotated soybean gene Glyma04g14361 [27], in this report designated as *GmaPPO12*. *GmaPPO12* is predicted to encode a protein with 601 amino acids. Plant polyphenol oxidases (PPOs) are enzymes that oxidize ortho-diphenols to *ortho*-diquinones, or convert monophenols to ortho-diphenols. They are often considered to be defense proteins due to their pathogen and wound-induced expression [26].

### *GmaPPO12* is an immediate-early *P* *. sojae* infection response gene

**Figure 2:**
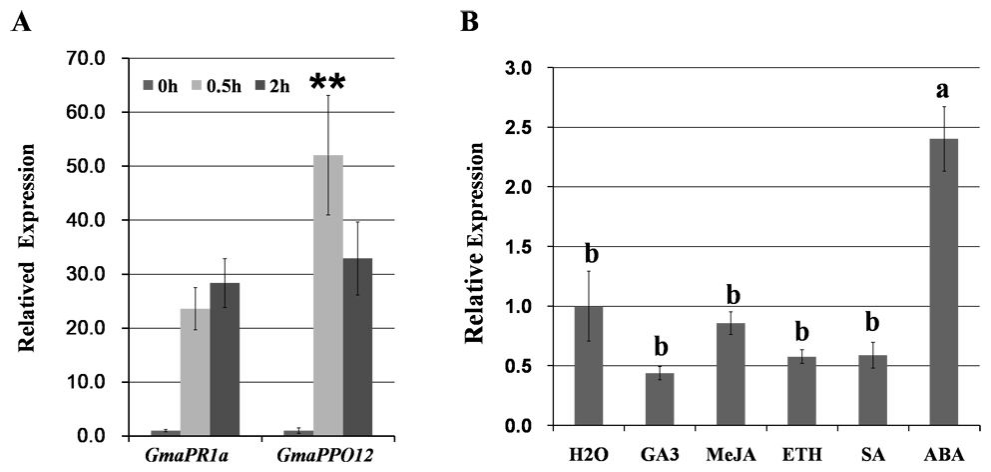
Induced expression of *GmaPPO12* and *GmaPR1a* in soybean roots. A. Expression patterns of soybean *GmaPPO12* and *GmaPR1a*. Quantitative RT-PCR analysis was carried out to detect *GmaPPO12* and *GmaPR1a* transcript abundance in 7-day-old soybean roots submerged in suspensions containing 10^5^ zoospores of *P* *. sojae* per milliliter at 0 hpi, 0.5 hpi and 2 hpi (hours post inoculation). The soybean *ACT20* gene was used as a reference gene with designed primers shown in Table S1. Transcript levels represent *GmaPPO12* or *GmaPR1a* mRNA levels compared with reference mRNA levels and then normalized to the treated roots at 0 hpi. Biological triplicates were averaged. A significant difference compared using Student’s t-test (** for p < 0.01). Bars indicate the standard error. B. Effects of the tested hormones on accumulation of soybean *GmaPPO12* gene transcripts. Quantitative RT-PCR analysis was carried out to detect *GmaPPO12* transcript abundance in 7-day-old soybean roots. The roots were submerged in ddH_2_O (control) or JA (100 µM), SA (100 µM), MeJA (100 µM), GA_3_ (100 µM), ethephon (100 µM) for 3 h. The soybean *ACT20* gene was used as a reference gene. Each column represents the relative transcript expression to the soybean reference gene then normalized to the roots treated with ddH_2_O. Biological triplicates were averaged. Means with different letters at the top are significantly different (p < 0.05 Duncan’s multiple range tests), lines designated with the same letter exhibit no significant difference in response to the tested hormones. Bars indicate the standard error.

To further validate that the *GmaPPO12* gene is an immediate-early *P* *. sojae* infection response gene, we used quantitative reverse transcription-polymerase chain reaction (RT-PCR) to characterize its expression profile in soybean roots at 0.5 and 2 h after infection with *P* *. sojae* zoospores. Since the *GmaPPO12* gene has three other highly conserved homologs, we designed a pair of specific primers to distinguish it from the others (Figure S1). Figure 2A shows that the transcript levels of *GmaPPO12* increased at 0.5 hpi and 2 hpi and showed 53-fold and 33-fold higher mRNA levels, respectively, compared to 0 hpi. The transcript levels of soybean *PR1a*, a known pathogen-inducible gene, also increased at 0.5 hpi and 2 hpi and showed 23-fold and 28-fold induction, respectively compared to 0 hpi (Figure 2A). Our results show that the induction levels of *GmaPPO12* are much higher than those of *PR1a* at 0.5 hpi (p < 0.01). Thus, we selected this gene for further analysis and designated its promoter as a *P* *. sojae* immediately induced promoter.

### *GmaPPO12* promoter contains many known *cis*-elements and is not up-regulated by the tested hormones except for ABA

A 1,500-bp fragment harboring the -1500 to +1 region (transcription start site of *GmaPPO12* as +1) was isolated from soybean as the candidate pathogen-induced promoter. This fragment (*GmaPPO12* promoter) was submitted to PlantCARE [28] to detect the *cis*-regulatory elements. Several elements that could be classified into three groups were identified within this promoter (Figure S2). The first group is the basal regulatory elements, including nine CAAT-box elements [29]. The second group includes the well studied pathogen-inducible *cis*-acting elements, including six GT-1 elements [30], two W-box elements [31], three WRKY-binding site WRKY elements [30], eight R response MYC binding sites [32], one regulatory defense-related gene CACGTG motif [33]. The third group is involved in abiotic stress or hormone response, such as the two GA-responsive GARE elements [34], one central element of gibberellin response MYB [35], one auxin response factor (ARF) binding sequence SURE [36], three water stress responsive elements MYB2 elements [37], one transcriptional activator ARR1AT [38], three abscisic acid (ABA) -responsive complex RYR elements [39], four ABA or stress responsive MYB1AT elements [40], one light-activated transcription binding site T-box [41], and one plant bZIP protein binding site SUREP [42]. In conclusion, based on the predictive identification of putative *cis*-elements, we hypothesize that the transcriptional activity of *GmaPPO12* is induced by different biotic and abiotic stresses.

Since several predicted hormone-responsive elements exist in the *GmaPPO12* promoter region, we used quantitative RT-PCR to examine the effects of five plant hormones on the *GmaPPO12* gene (Figure 2B). We harvested the 7-day-old soybean roots and washed them with water, then immersed them in jasmonic acid (JA), salicylic acid (SA), abscisic acid (ABA), ethephon (ETH), gibberellic acid (GA), or double-distilled water (ddH_2_O) (control) for 3 h. The results show that the transcript levels of *GmaPPO12* was slightly reduced when treated with JA, SA, ethephon, or GA3, and have a 2.4-fold increase occurred when treated with ABA (p < 0.05). Thus, *GmaPPO12* is not up-regulated by the tested hormones except for ABA.

### The *GmaPPO12* promoter mediates rapid and strong GUS expression upon *Phytophthora capsici* infection in *N benthamiana* leaves

**Figure 3:**
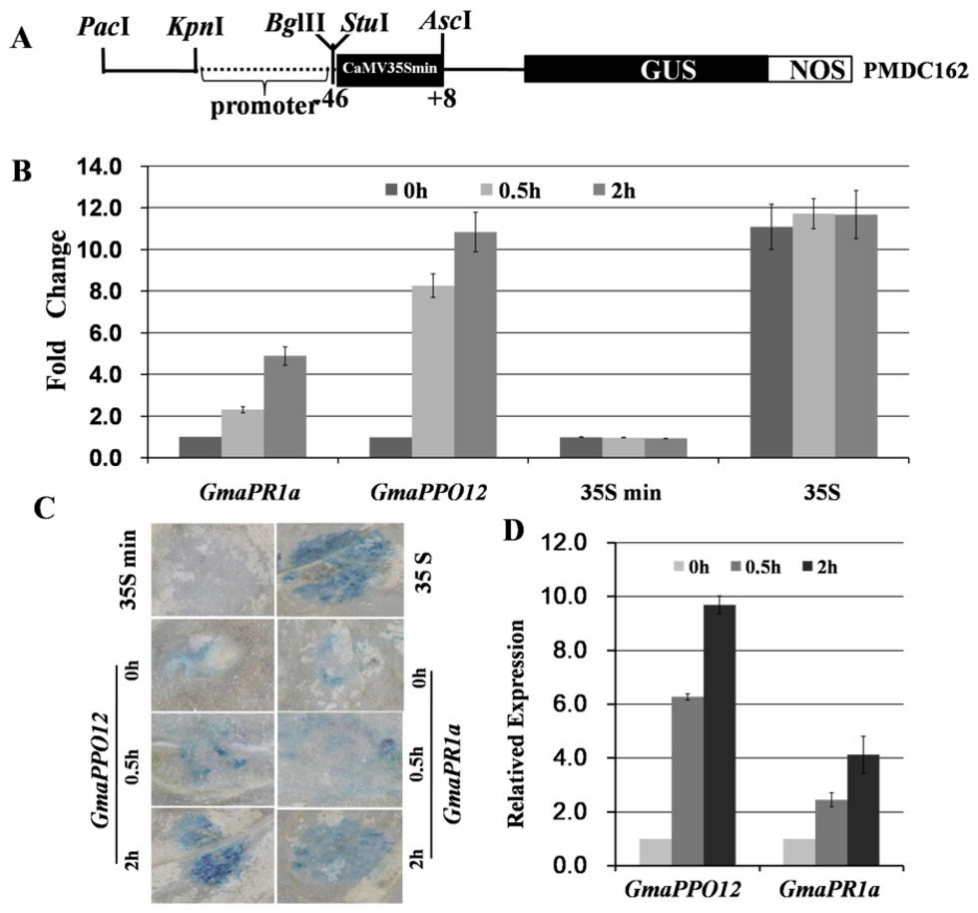
Induction of the *GmaPPO12 and GmaPR1a* promoters upon *Phytophthoracapsici* infection in *Nicotiana* *benthamiana* leaves. A. Scheme of the pathogen-induced promoter GUS fusion constructs. The studied promoters were inserted between the *Kpn*I and *Bgl*II sites in pMDC162 upstream of the -46 35S promoter of the *Cauliflower mosaic virus* (CaMV 35S). B. Enzymatic assay of GUS activity in expanded 6-week-old *N* *. benthamiana* leaves. The leaves were infiltrated with *Agrobacterium* harboring *GmaPPO12* or *GmaPR1a* promoter: GUS constructs, and then submerged in *P* *. capsici* zoospores 3 days after agroinfiltration. GUS activity levels were measured by ﬂuorimetric assays in protein extracts from treated leaves at 0 hpi, 0.5 hpi and 2 hpi. Each column represents the fold change of *P* *. capsici* induction at 0.5 hpi and 2 hpi to 0 hpi and normalized to 35S min. Labels are as follows: *GmaPR1a* promoter; *GmaPPO12* promoter; 35S minimal promoter; 35S full-length promoter fragments. Experiments were in triplicate and error bars show the standard error. C. Histochemical GUS staining of *GmaPPO12* and *GmaPR1a*: GUS constructs in *N* *. benthamiana* leaves. The leaves were treated as in B. GUS activity increased dramatically at *GmaPPO12* and *GmaPR1a* construct agroinfiltrated regions at 0.5 hpi and 2 hpi. No visible GUS activity was noted at 35S min agroinfiltrated regions. GUS activity was relatively strong at 35S (CaMV 35S full-length promoter fragments) agroinfiltrated regions. D. GUS reporter gene transcript levels at different time points post inoculation measured by quantitative RT-PCR. Total RNA was extracted from transiently expressing *N* *. benthamiana* leaves. The leaves were treated as in B. Real-time RT-PCR analysis employed primers specific for GUS and the *N* *. benthamiana* actin gene is *EF1a*. Transcript levels represent the GUS mRNA levels compared with actin mRNA levels normalized to the roots treated at 0 hpi. Experiments were done in triplicate with error bars showing the standard error.

To determine the inducibility of the promoter, we generated a construct containing the whole promoter region (1,500-bp) fused with the *GUS* reporter gene along with a 35S minimal (-46-+8) region (Figure 3A). The 35S minimal region, 35S full-length promoter and soybean *PR1a* promoter were used as controls. We examined GUS expression levels using *Agrobacterium* -mediated transient expression assay in *N* *. benthamiana* leaves. The method is simple and efficient for the quantitative analysis of plant promoters *in vivo* [24,43].

We examined induction activity upon inoculation with *P* *. capsici* zoospores after the *Agrobacterium* strains were infiltrated into *N* *. benthamiana* leaves. Quantitative GUS enzyme activity assays demonstrated that *GmaPPO12* showed almost 8.2- and 10.8-fold induction, while *PR1a* showed 2.1- and 4.6-fold induction at 0.5 and 2 hpi (Figure 3B), respectively. Histochemical staining revealed that *GmaPPO12* showed stronger induction than *PR1a* by *P* *. capsici* , although untreated tobacco leaves containing *GmaPPO12* or *PR1a* showed weak background expression (Figure 3C). To further confirm the results, we used quantitative RT-PCR to measure GUS mRNA levels and found that the transcript expression of *GmaPPO12* was 6.1- and 9.5-fold at 0.5 and 2 hpi, and *PR1a* was 2.2- and 4.1-fold higher compared to uninfected tobacco leaves (Figure 3D), respectively. As expected, the leaves infiltrated with negative control (the construct only containing 35S minimal promoter) showed very low levels of GUS activity in all the three independent assays, while a full length 35S promoter fused to GUS exhibited high levels regardless of the treatment (Figure 3B-C). Importantly, the induction levels of GUS by *GmaPPO12* promoters were similar to the 35S promoter (Figure 3B-C), suggesting that the promoter is strongly and rapidly induced upon pathogen infection.

### The *GmaPPO12* promoter functions in soybean hairy roots

**Figure 4:**
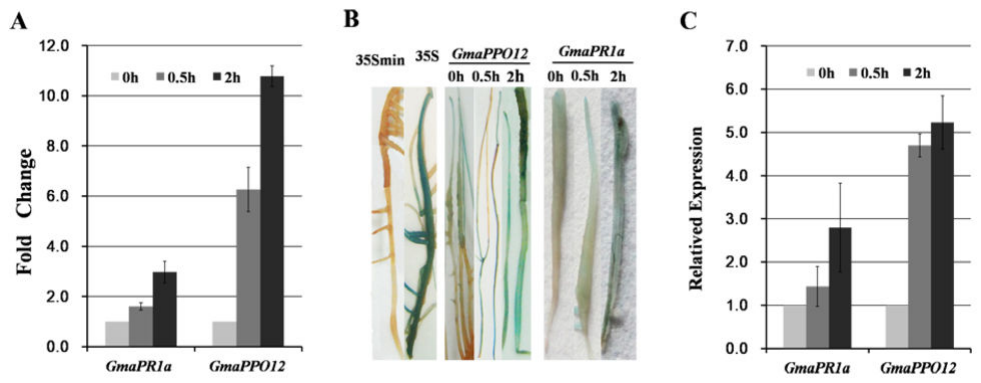
Validation of *GmaPPO12 and GmaPR1a* promoter function in soybean hairy roots. A. Enzymatic assay of GUS activity in multiple soybean hairy roots. The roots were inoculated with 10^5^ *P* *. sojae* zoospores, and analyzed at 0 hpi 0.5 hpi and 2 hpi. Each column represents the fold change in *P* *. sojae* induction at 2 hpi compared to 0 hpi and normalized to 35S min. Experiments were in triplicate, and error bars show the standard error. B. Histochemical staining of GUS activity in transgenic soybean hairy roots. Roots were treated as in A. No visible GUS activity in 35S min transgenic soybean hairy roots; GUS activity was relatively strong in 35S (CaMV 35S full-length promoter fragments) transgenic soybean hairy roots; GUS activity increased dramatically in *GmaPPO12* transgenic soybean hairy roots at 0.5 hpi and 2 hpi. C. *P* *. sojae* -induced expression analysis of the *GmaPPO12* promoter by quantitative RT-PCR. The expression levels of the *GUS* reporter gene in the multiple soybean hairy roots. Roots were treated as in A. The soybean *ACT20* gene was used as a reference gene. Transcript levels represent the *GUS* mRNA levels compared with soybean reference mRNA levels and then normalized to the hairy roots treated at 0 hpi. Experiments were done in triplicate with error bars showing the standard error.

To independently validate that *GmaPPO12* is an immediate-early *Phytophthora* infection gene, the vectors were introduced into soybean hairy roots by *Agrobacterium* -mediated transformation. The multiple soybean hairy roots were inoculated with *P* *. sojae* zoospores, and analyzed by histochemical staining, enzymatic assay, and quantitative RT-PCR. Enzymatic assay showed that *GmaPPO12* was induced 6.3- and 10.8-fold and *PR1a* was induced 1.6- and 3.0-fold at 0.5 and 2 hpi (Figure 4A), respectively. The histochemical staining shows that the GUS reporter activity driven by *GmaPPO12* was significantly higher than that of *PR1a* and non-treatment with *P* *. sojae* (0 hpi) in transgenic hairy roots (Figure 4B). we also used quantitative RT-PCR to measure GUS mRNA levels and found that the transcript expression of *GmaPPO12* was 4.8- and 5.2-fold increases at 0.5 and 2 hpi, respectively, compared to that of *PR1a* with 1.4- and 2.8-fold increases at 0.5 and 2 hpi (Figure 4C), respectively. These results demonstrate that the *GmaPPO12* promoter, compared to the *PR1a* promoter, was more strongly induced upon infection in soybean.

### Deletion analysis identifies an important responsive region in the promoter

**Figure 5:**
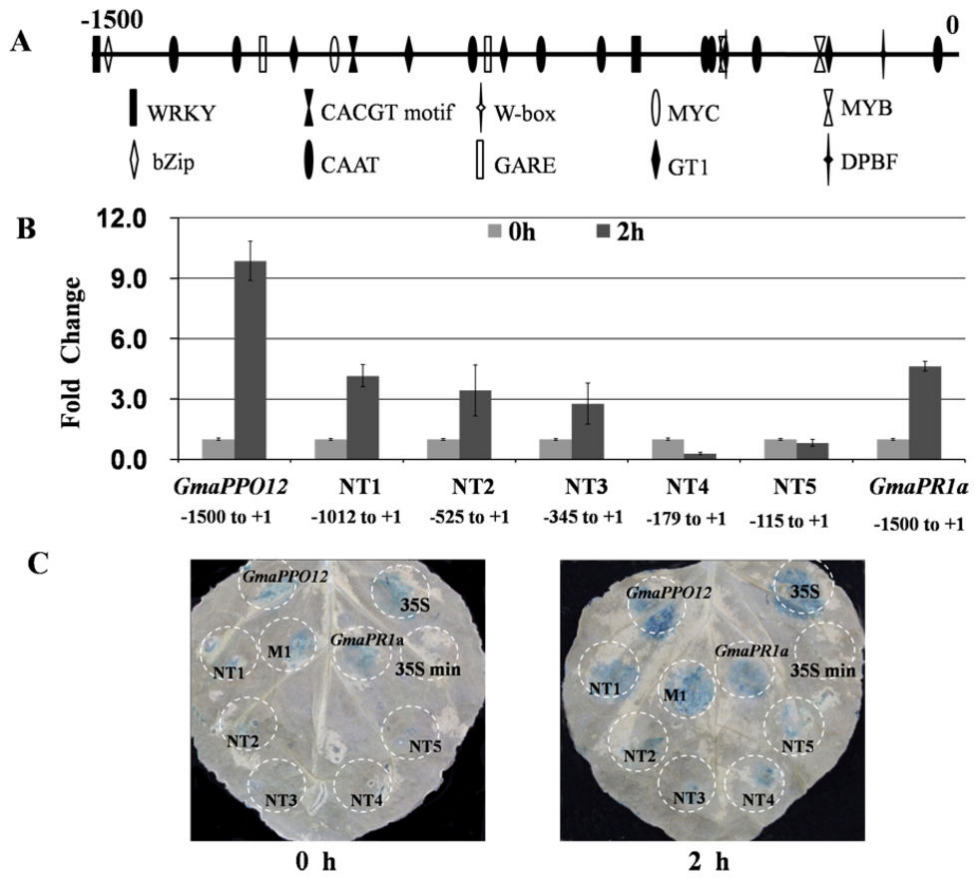
N-terminal deletion analysis of the *GmaPPO12* promoter in *Nicotiana* *benthamiana* leaves. A. Structural mapping of the regulatory elements of the *GmaPPO12* gene promoter. B. Enzymatic assay of N-terminal deletion constructs in *Nicotiana* transiently expressing leaves. The expanded 6-week-old *N* *. benthamiana* leaves were infiltrated with *Agrobacterium* harboring the constructs, and inoculated with *Phytophthoracapsici* zoospores 3 days after infiltration. Enzymatic assay at 0 hpi and 2 hpi. Each column represents the fold change of the *P* *. capsici* induction at 2 hpi compared to 0 hpi and normalized to 35S min. Labels are as follows: *GmaPPO12* promoter; N-terminal deletion mutants: NT1 (-1012 to +1), NT2 (-525 to +1), NT3 (-345 to +1), NT4 (-179 to +1), NT5 (-115 to +1); *GmaPR1a* promoter. Experiments were in triplicate, and error bars show the standard error. C. Histochemical GUS staining of different constructs in *Nicotiana* transiently expressing leaves at 0 hpi and 2 hpi. The leaves were treated as in B. No visible induced GUS activity was detected at agroinfiltrated regions, including NT2, NT3, NT4, NT5, 35S min; GUS activity increased dramatically at the agroinfiltrated regions, including *GmaPPO12*, NT1, M1, *GmaPR1a*; GUS activity was continuously high at agroinfiltrated regions, including 35S (CaMV 35S full-length promoter fragments).

To locate the pathogen-responsive *cis*-regulatory regions, a series of progressive 5’ or 3’ truncated *GmaPPO12* promoters were fused to a minimal 35S promoter and the *GUS* reporter gene (Figure 5A-S3). In *Agrobacterium* -mediated transient expression of *N* *. benthamiana* leaves, the potential expression patterns and pathogen-inducible activities of those promoter mutations were evaluated by histochemical staining and enzymatic assay after treatment with *P* *. capsici* zoospores for 2 h. Truncation of the promoter to -525 (NT2) significantly reduced GUS enzyme activities (Figure 5B-C). Deletions beyond this position to -179 (NT4) resulted in no detectable GUS activity and transcription levels. Truncation of the promoter to -1012 (NT1) reduced the GUS enzyme activity to the same levels as the native *GmaPR1a* promoter (Figure 5B-C).

**Figure 6:**
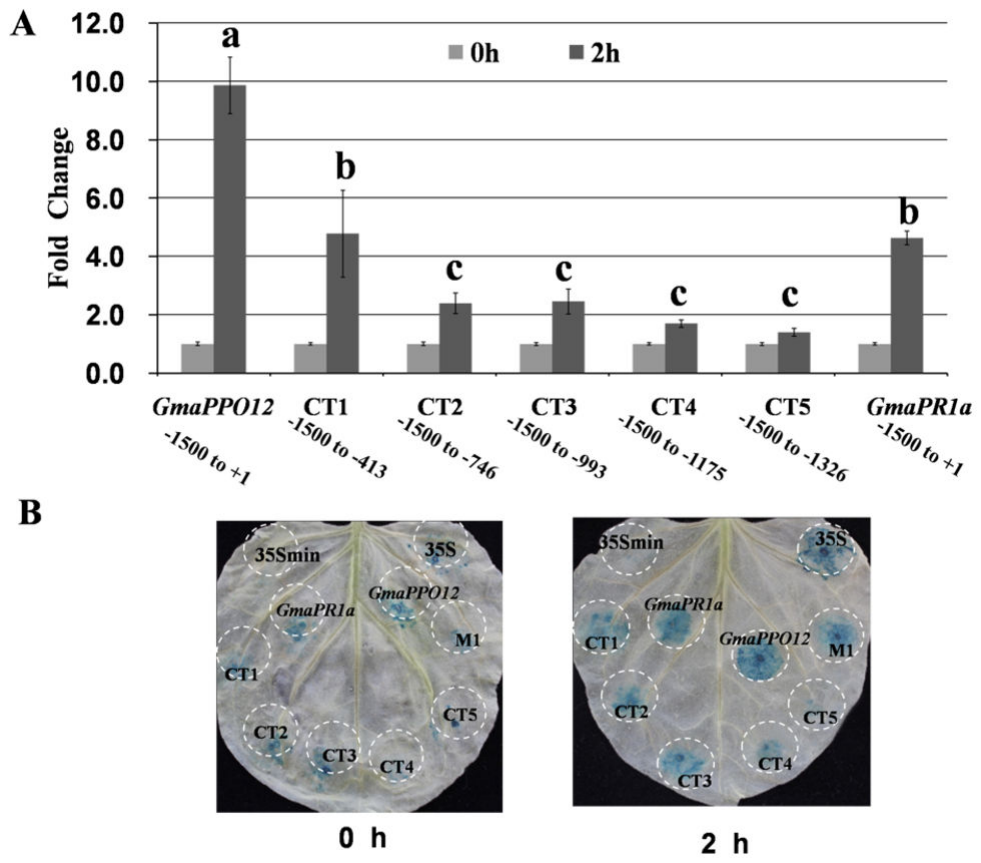
C-terminal deletion analysis of *GmaPPO12* promoter activity in transiently expressing *Nicotiana* *benthamiana* leaves. A. Enzymatic assay of C-terminal deletion constructs in *Nicotiana* transiently expressing leaves. The expanded 6-week-old *N* *. benthamiana* leaves were infiltrated with *Agrobacterium* harboring constructs and inoculated with *Phytophthoracapsici* zoospores 3 days after infiltration. Enzymatic assay at 0 hpi and 2 hpi. Each column represents the fold change in *P* *. capsici* induction at 2 hpi compared to 0 hpi and normalized to 35S min. Labels are as follows: *GmaPPO12* promoter; C-terminal deletion mutants: CT1 (-1500 to -413), CT2 (-1500 to -746), CT3 (-1500 to -993), CT4 (-1500 to -1175), CT5 (-1500 to -1326); *GmaPR1a* promoter. Experiments were performed in triplicate. Means that have different letters at the top are significantly different (p < 0.05 Duncan’s multiple range tests), lines designated with the same letter exhibit no significant difference of the GUS enzyme activity. Error bars show the standard error. B. Histochemical GUS staining of different constructs in *Nicotiana* transiently expressing leaves at 0 hpi and 2 hpi. Leaves were treated as in A. No visibly induced GUS activity was detected at agroinfiltrated regions, including 35S min; GUS activity was relatively weak at agroinfiltrated regions, including CT4, CT5; GUS activity increased dramatically at agroinfiltrated regions, including CT1, CT2, CT3, M1, *GmaPPO12, GmaPR1a*; GUS activity was continuously high at agroinfiltrated regions, including 35S (CaMV 35S full-length promoter fragments).

A series of progressive 3’ deletions of Gma*PPO12* were evaluated. The deletion of the promoter sequence up to -1087 (CT1) reduced the GUS enzyme activity to around half of the native promoter. For other four progressive deletions up to -751 (CT2), -507 (CT3), -325 (CT4) and -174 (CT5), the GUS enzyme activity were significantly reduced (p < 0.05) (Figure 6A). These results were also confirmed by a histochemical staining assay (Figure 6B). Thus, 5’ and 3’ deletions together suggest that the fragment from -1012 to -413 is essential for promoter activity, although the fragment from -1500 to -1012 is partially required in response to pathogen inoculation.

### A 113-bp fragment is a vital pathogen-induced region in *GmaPPO12* promoter

**Figure 7:**
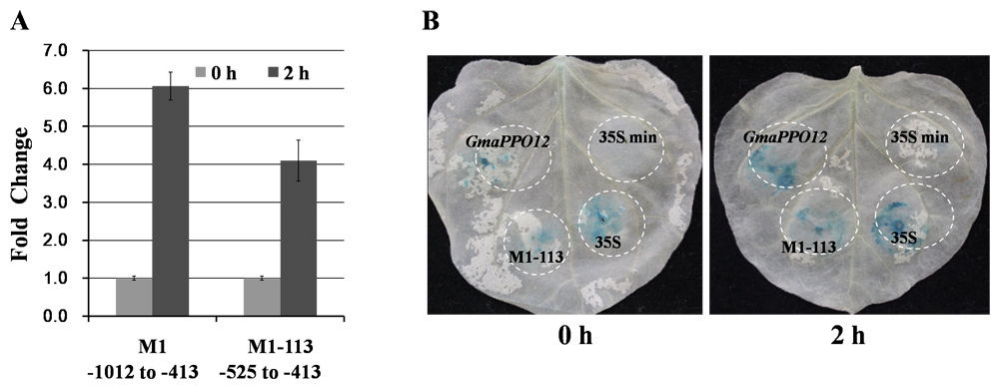
Functional analysis of two fragments in the promoter. A. Enzymatic assay of GUS activity in transient *Nicotiana* *benthamiana* leaves. Expanded 6-week-old *N* *. benthamiana* leaves were infiltrated with *Agrobacterium* harboring the deletion constructs M1 (-1012 to -413) and M1-113 (-525 to -413). Leaves were inoculated with *P* *. capsici* zoospores 3 day after infiltration and assayed at 0 hpi and 2 hpi. Each column represents the fold change from the *P* *. capsici* induction at 2 hpi compared to 0 hpi and normalized to 35S min. Labels are as follows: M1 (-1012 to -413) and M1-113 (-525 to -413). Experiments were in triplicate, and error bars show the standard error. B. Histochemical GUS staining of different constructs in *Nicotiana* transiently expressing leaves at 0 hpi and 2 hpi. Leaves were treated as in A. No visible induced GUS activity was detected at the injection region in 35S min. GUS activity was relatively weak at the injection region in M1-113. GUS activity was increased dramatically at the injection region of *GmaPPO12*. GUS activity was continuously high at agroinfiltrated regions for 35S (CaMV 35S full-length promoter fragments). Experiments were done in triplicate with error bars showing the standard error.

To further confirm the activity of the fragment from -1012 to -413 (M1), we generated a plant expression vector containing the fragment (Figure S3). In *N* *. benthamiana* leaves, GUS activity increased 6.0-fold upon induction by *P* *. capsici* zoospore infection for 2 h (Figure 7A). Histochemical staining showed concordant results (Figures 5C-6B). The results support that the fragment from -1012 to -413 (M1) is essential to confer promoter activity in response to induction.

On the basis of our above results, we found that a 113-bp fragment from -525 to -413 was shared by three analyzed mutants, they are M1 (-1012 to -413), NT1 (-1012 to +1), and CT1 (-1500 to -413), which showed high pathogen-induced activity (Figures 5-6). We then measured activity of the fragment. In *N.* benthamiana leaves, the enzymatic assay showed 4.1-fold induction upon infection (Figure 7A). An obvious induction was also observed in histochemical staining assays (Figure 7B). This 113-bp fragment contains two basal regulatory CAAT-box elements [29], two ABA or stress responsive MYB1AT elements [40], three R response MYC elements [32], and one bZIP transcription factor binding core DPBF sequence [44] (Figure S3). Thus, the results suggest that the 113-bp sequence from -525 to -413 can mediate induction activity at around 40% compared to activity of the full length promoter.

## Discussion

We are unaware of pathogen-inducible promoters have been identified and characterized in soybeans. Here, we screened the published microarray data [25] and found that the *GmaPPO*12 gene is strongly induced by *P* *. sojae* . We elucidated its expression pattern by quantitative RT-PCR analysis in soybeans, transient expression in *N* *. benthamiana* , and stable expression in soybean hairy roots. We found that the gene had three expressional characterizations. First, it is an immediate-early pathogen response gene because it can be induced by *P* *. sojae* infection at 0.5 h in soybean, and its promoter also can mediate fast induction activity in *N* *. benthamiana* by *P* *. capsici* . Second, its induction is strong. The expression levels upon infection are higher than that of the *GmaPR1a* gene in soybean. Third, the expression is specific to infection. It cannot be induced by the tested hormones in soybean, and its background expression is almost undetectable in soybean and *N* *. benthamiana* . This promoter could be used in transgenic plants to enhance their resistance to *Phytophthora* and perhaps other oomycete diseases. Finally, we identified a small responsive region by progressive 5’ and 3’ deletions. The small functional segment could serve as a candidate to further identify novel pathogen responsive *cis*-elements or to produce synthetic pathogen-inducible promoters.

The gene is predicted to encode a member of the polyphenol oxidase (PPO) family that catalyzes the oxidation of phenolics to quinones [26,45]. In green plants, the *PPO* gene family is highly variable in both gene number and structure, except in the genus *Arabidopsis* [26]. The enzymes play many divergent roles in a variety of processes, including defense processes [46,47]. In tomatoes, for example, PPO overexpression increased resistance to *Pseudomonas syringae* and several insects [45]. PPOs are also implicated in latex coagulation [48], and the biosynthesis of aurones and lignans [49,50]. Soybean contains 11 PPO isoforms [26], which correlate with its abundant flavonoids. Here, we showed that *GmaPPO12* was rapidly and strongly induced by *P* *. sojae* infection. However, the role of *GmaPPO12* in disease resistance still needs to be addressed.

Patterns of gene expression in soybean upon *P* *. sojae* infection have been investigated in several independent studies [25,51,52]. Only 22 soybean genes were identified as upregulated early in infection (3 hrs). The genes included *GmaPR1a* and a gene encoding flavin adenine dinucleotide (FAD)-linked oxidoreductase [51]. Here, we identified *GmaPPO12* as being strongly inducing at 0.5 h postinfection. Its fold change was higher than that of *GmaPR1a*. Characterization of the *GmaPR1a* promoter in *N* *. benthamiana* leaves and soybean hairy roots also supported the idea that the gene was rapidly and highly induced by *Phytophthora* pathogens. Moreover, the fold change in *GmaPPO12* promoter driven GUS expression was lower than that of the endogenous gene in soybean. Quantitative RT-PCR analysis showed that the gene was upregulated 53-fold at 0.5 hpi in soybean, but the *GUS* gene was only induced 6.1-fold in *N* *. benthamiana* and 4.8-fold in soybean hairy roots, respectively. This difference may be due to that the stress-induced expression of the endogenous gene driven by the native promoter not being similar to the heterologous *GUS* gene fused with the same promoter, as observed in previous studies [53]. We have noted that the promoter driving GUS expression exhibited unwanted background expression, although the levels were low. One possible reason is that the agroinfiltration associated wounding induced gene expression. Further studies will be required to test the utilization of the cloned promoter to drive functional disease resistance genes.

Promoter deletion analysis revealed that two fragments appeared to be partially sufficient for the induction activity of the *GmaPPO12* promoter. The 599-bp and 113-bp fragments accounted for over 60% and 40%, respectively compared to activity of the full length promoter. The identified smaller fragment contained several putative *cis*-elements that are involved in defense responses, including two MYB binding sites, three MYC binding sites, and one DPBF-box. The MYB binding sites have been identified in the parsley *PAL1* promoter as the sites of fungal elicitor-inducible DNA–protein interactions [54], and the tobacco *PR1a* promoter [55]. The MYC recognition sites play a critical role in MYC regulation of innate immune responses against mycobacterial infection [56]. Testing these elements from soybeans is now a priority so that we can design the synthetic promoters that are specific and highly induced by pathogens.

## Materials and Methods

### Plant materials and inoculation

*Nicotiana* *benthamiana* was used for transient expression and soybean (cv. Williams) was used to provide stable transgenic soybean hairy roots. The plant seeds were grown in a growth chamber at 25°C under fluorescent white light in a 16: 8-h light/dark cycle. Seven-day-old soybean roots or soybean hairy roots (see below for the methods to obtain transgenic hairy roots) were inoculated with suspensions containing 10^5^ zoospores of *P* *. sojae* (Strain, P6497) per milliliter. Six-week-old *N* *. benthamiana* leaves were immersed in suspensions containing 10^5^ zoospores of *P* *. capsici* [57] 72 h after agroinfiltration. The samples were collected at 0.5 and 2 h, and non-treated tissues were used for control. For hormone treatments, 7-day-old soybean roots were submerged in different hormones for 3 h, and the concentration of hormones is jasmonic acid (100 µM), salicylic acid (100 µM), abscisic acid (100 µM), ethephon (100 µM), gibberellic acid (100 µM), respectively. The control plants were immersed in water. For each treatment and time point, three leaves or roots from three individual plants were collected for duplications.

### Construction of the transformation vector

Genomic DNA was extracted from 6–7-day-old soybean roots, following the cetyltrimethylammonium bromide (CTAB) isolation procedure [58]. The *GmaPPO12* and *GmaPR1a* promoters were isolated with the designed primers (Table. S1). The obtained promoters cover a sequence 1,500-bp in length upstream of the transcription start site. Five 5’ deletions, five 3’ deletions, and two 5’ and 3’ deletions of the *GmaPPO12* promoter region were generated by PCR, with forward primers containing a *Kpn*I restriction site and a reverse primer containing a *Bgl*II restriction site. The sequences of promoters used for the amplification of the deletion promoter fragments are summarized in Figure S3. All of the promoter fragments were digested with *Kpn*I and *Bgl*II and cloned into the *Kpn*I-*Bgl*II sites of pMDC162 [59], a plant expression vector fused with the CaMV 35S minimal promoter [60] and *GUS* reporter gene. A full description of plasmid construction can be found in Figure 3A. The pMDC162 vector containing the CaMV 35S minimal promoter lacking *cis*-acting elements was constructed and used as a negative control. PBI121 contains the *GUS* (or *uidA*) gene under the control of the CaMV 35S promoter as the positive control. Both plasmids contain the selectable marker gene *nptII*, which confers resistance to kanamycin. All primers used in this study are listed in Supplemental Table. 1. All plasmids containing expression cassettes were confirmed by sequence analysis and introduced into *Agrobacterium* strains by electroporation for transformation.

### Agroinfiltration of *N benthamiana* leaves

All the constructs were introduced into *Agrobacterium tumefaciens* strain GV3101 by electroporation, as described by Ainsworth et al [61]. Agroinfiltration of *N* *. benthamiana* leaves was based on the methods described in Llave [62] with the following modifications. Individual agrobacteria colonies were grown on Luria-Bertani plates with kanamycin (50 µg mL^-1^) for 48 h at 28°C. A single positive colony was used to inoculate a 5 mL culture (LB with 50 µg mL^-1^ kanamycin). Bacteria were pelleted, resuspended in infiltration medium [10 mM MgCl_2_, 10 mM MES, 150 µM acetosyringone (pH 5.6)] to an OD_600_ of 0.5-0.6, then incubated at room temperature for 3 h. The bacterial suspension was infiltrated into the abaxial side of fully expanded 6-week-old *N* *. benthamiana* leaves using a needleless 1-mL syringe. For each experiment, the positive control (PBI121 intron with 35S-GUS), the negative control (TATA-GUS in pMDC162 intron), and the constructs under investigation were infiltrated in areas of the same leaf or different leaves. After infiltration, the plants were kept in the greenhouse 72 h for inoculation.

### Soybean hairy root transformation

The introduction of expression constructs into soybean hairy roots using the *Agrobacterium* *rhizogene* K599 cucumopine strain [63] was by electroporation as described by Ainsworth et al [61]. Individual colonies were picked and verified by enzyme restriction analysis of DNA extracted from randomly selected clones. Plant inoculation was conducted according to Savka [63] and modified as follows: soybean seeds were surface-sterilized with chlorine gas and germinated on Murashige and Skoog (MS) culture medium for 5-6 days in a 16: 8-h light/dark cycle incubator at 25°C. Cotyledons were wounded on the abaxial side with scalpel blades dipped in a culture of *Agrobacterium* *rhizogenes* K599 harboring the different constructs and cultured abaxial-side-up on MS culture medium. Carbenicillin and Cefotetan 250 µg/mL were added to inhibit the growth of *A* *. rhizogenes* and incubated at 25°C in the dark. The putative transformed hairy roots about 18-20 days after root emergence and at lengths of 4- to 6-cm were used for inoculations.

### Histochemical and ﬂuorometric GUS assays

Expression of the GUS reporter was measured at 0 and 2 h after zoospore treatment. Both histochemical and ﬂuorometric GUS assays were based on methods described by Jefferson [64] with the following modifications. For histochemical staining, the plant tissues were incubated at 37°C overnight (12 h) in the dark in 1 mM X-Gluc (5-bromo-4-chloro-3-indolyl-b-D-glu-curonide) in 100 mM sodium phosphate (pH 7.0), 10 mM EDTA, 0.5 mM potassium ferricyanide, 0.5 mM potassium ferrocyanide, 0.3% (v/v) Triton X-100, and 20% (v/v) methanol to eliminate endogenous GUS activity [65]. After 12 h staining, tissues were destained in an ethanol series (50, 70, and 95%) to remove chlorophyll, stored in 70% (v/v) ethanol, and photographed with a digital camera.

For ﬂuorometric assays, the plant tissues were homogenized in 1 mL extraction buffer [50 mM NaH_2_PO_4_, pH 7.0, 10 mM EDTA, 0.1% Triton X-100, 0.1 (w/v) sodium laurylsarcosine, 10 mM β-mercaptoethanol], and centrifuged at 12,000*g* for 10 min at 4^°^C. Total protein in tissue homogenates was quantiﬁed by the Bradford [66] method, using bovine serum albumin (BSA) as a standard. The supernatant (40 µl) was mixed with 400 µL 2 mM 4-methylumbelliferyl-β-d-glucuronide (MUG) on ice and 100 µL was transferred immediately to a fresh tube containing 900 µL of GUS stop buffer (0.2 M Na _2_CO_3_) to serve as a control. GUS assays were performed at 37^°^C for 30 min. Stop buffer (0.2 M Na _2_CO_3_) and 50 nM to 1 mM 4-methylumbelliferone (4-MU) were used for calibration and standardization. Fluorescence (excitation 365 nm, emission 455 nm) of each sample was determined in a SpectraMax M5 (Molecular Devices, Sunnyvale, CA, USA), and the GUS activity calculated from the slope of the best fit line through the three points to give the increase in fluorescence per minute, and expressed as nanomoles of 4-methyl-umbelliferone (4-MU) produced per minute per milligram of soluble protein. Duncan’s multiple range tests were used for statistical analysis (p < 0.05) (Figure 6A).

### Quantitative RT-PCR

Total RNA was extracted by using the PureLink RNA mini kit (Invitrogen), and ﬁrst strand cDNA was synthesized using an iScript cDNA Synthesis kit (*Takara Bio DRR036A*). cDNAs were diluted to 100 ng/µL and combined with SYBR master mix. Expression of each gene was normalized to a reference gene of either the soybean elongation factor gene (Genbank accession no. XM_003531518.1) or *Nicotiana tabacum* elongation factor 1-alpha mRNA (Genbank accession no. AF120093.1), and the speciﬁc primers named ACT20-F, R, or EF1a-F, R (see Supplemental Table 1). PCR was performed in triplicate using 96-well optical reaction plates. Quantitative RT-PCR thermal cycler conditions and reaction mixtures were according to the manufacturer’s instructions (SYBR Premix Ex Taq^TM^). The ﬂuorescence threshold value and gene expression data were calculated using the (ABI 7500 SDS) system software. The endpoint was used in the real-time PCR quantification [67], Ct is defined as the PCR cycle number that crosses an arbitrarily placed signal threshold. The level of transcript abundance relative to the reference gene (termed △Ct) was determined according to the function △Ct =Ct (test gene) - Ct (reference gene). To compare untreated and treated expression levels, the function △△Ct was ﬁrst determined using the equation △△Ct = △Ct (treatment) -△Ct (control) (where control represented mock-treated plants). The induction ratio of treatment/control was then calculated by the equation 2^(-△△Ct). The primer sequences are listed in Supplemental Table 1. Student’s t-tests (p < 0.01) (Figure 2A) or Duncan’s multiple range tests (p < 0.05) (Figure 2B) were used for statistical analysis.

### Phylogenetic tree and promoter analysis

A Phylogenetic tree of soybean PPO proteins was constructed by the neighbor-joining method using MEGA 4.0 [68,69]. Genetic distances were estimated using the Dayhoff amino acid substitution matrix. Positions in the alignment lacking amino acid residues were excluded from the pairwise distance estimates. Bootstrap replicates (1000) were used to indicate the level of support from the data for each node of the tree. Predictions pertaining to the types of introns were independently checked using CIWOG [70]. N-terminal transit peptide sequences were predicted using ChloroP 1.1 and TargetP 1.1 [71,72]. The PPO protein conserved regions were analyzed using a combination of NCBI BLASTP and SMART (Simple Modular Architecture Research Tool, http://smart.embl-heidelberg.de/).

The *cis*-regulatory elements in the promoters were predicted using PlantCARE (http://bioinformatics.psb.ugent.be/webtools/plantcare/html) [28].

## Supporting Information

Table S1 Polymerase chain reaction (**PCR**) primers used in this **study**.
(DOC)

Figure S1 Neighbor-joining phylogenetic tree of soybean PPOS, together with corresponding visual representation of conserved regions, functional motifs, and relative intron positions. The ClustalW multiple sequence alignment was formed using the deduced soybean PPO proteins. The tree was constructed from the ClustalW alignment using the neighbor-joining method of the MEGA program. The scale bar represents 0.05 substitutions per site and the numbers next to the nodes are bootstrap values from 1,000 replicates. Predicted targeting sequences are colored green (chloroplast transit peptide), black (signal peptide), or gray (unknown). The CuA and CuB domains are colored blue, and C-terminal conserved areas are dark gray. Approximate intron positions are shown as vertical bars, mapped onto the predicted protein.
(TIF)

Figure S2 Nucleotide sequence of the soybean *GmaPPO12* gene. The translational start sites (+1) are shown in red. Upstream of the translation start sites (+1) is the promoter sequence. The promoter motifs with significant similarity to the previously identified *cis*-acting elements are shaded and the names are given under the elements. The translation initiation codon (M) downstream amino acid sequence contains PPO conserved regions: the N-terminal chloroplast transit peptide sequence is colored green, CuA and CuB domain sequences are colored blue, C-terminal DWL and KFDV domains are colored red, and unknown sequences are colored gray. The nucleotide sequences colored blue-green are microarray EST. The sequence in purple boxes indicates the primers for quantitative RT-PCR to detect the inducibility of *GmaPPO12*.
(TIF)

Figure S3 The overall results of the deletion mutant analysis of the soybean *GmaPPO12* promoter. Structural mapping of the deletion mutations containing different *cis*-elements are presented schematically with a line representing the portion of sequence that was not deleted. The position of the last remaining base of the promoter sequence for each mutant is indicated on the left. The enzymatic activity upon infection of the corresponding deletion mutations is shown at the right.
(TIF)
