# Supplementary material for: Identification and Functional Characterization of the Soybean GmaPPO12 Promoter Conferring Phytophthora sojae Induced Expression
Source: PLoS One. 2013 Jun 28;8(6):e67670. doi: 10.1371/journal.pone.0067670 (PMC3695865; doi:10.1371/journal.pone.0067670)
Supplement: Table S1 — study. (DOC) [file pone.0067670.s001.doc]

**Supplemental Table 1. Polymerase chain reaction (PCR) primers used in this study**.

| Primer name | Primer sequence (5’ to 3’)^a, b, c^ | Purpose |
| --- | --- | --- |
| PR1-F | GG**GGTACC**GGTACATATCAAGTTCGTAC | *GmaPR1a* Promoter clone |
| PR1-R | GA*AGGCCT*CTTAATGCACATGTACCCCAT | *GmaPR1a* Promoter clone |
| P_1_-F | GG**GGTACC**TGATGACAAAAAGATACATGC | *GmaPPO12* and 3’deletion analysis clone |
| P1-R-1 | TTATATAGAGGAAGGGTCTTGCGAAGGCCTTAGATCTGCCCCCAGGAGCATCCCAGTT | *GmaPPO12* and 35S min Promoter clone |
| P1-R-2 | GGCGCGCCCAGCGTGTCCTCTCCAAATGAAATGAACTTCCTTATATAGAGGAAGGGT | *GmaPPO12* and 35S min Promoter clone |
| P_1_T_1_-F | GG**GGTACC**CTCATGAGAGGGCTTTCGACGA | 5’deletion analysis and mutant M1 |
| P_1_T_2_-F | GG**GGTACC**GAACATTCTACTTGGCCTAG | 5’deletion analysis |
| P_1_T_3_-F | GG**GGTACC**GATTTCAAGTTCCCTCCCTCCA | 5’deletion analysis |
| P_1_T_4_-F | GG**GGTACC**ATTGCGACGGTGGATATCAC | 5’deletion analysis |
| P_1_T_5_-F | GG**GGTACC**GCTCTTCTTTCCTTACCACAG | 5’deletion analysis |
| P_1_T-R | GAAGATCTGCCCCCAGGAGCATCCCAGTTCCAAAAT | 5’deletion analysis and mutant M1 |
| P_1_R_1_-R | GAAGATCTCACATGTGGTTAGGTCTGGA | 3’deletion analysis |
| P_1_R_2_-R | GAAGATCTTTAGACCCTTTCAGCCCT | 3’deletion analysis |
| P_1_R_3_-R | GAAGATCTTCGAAAGCCCTCTCATGAGACT | 3’deletion analysis |
| P_1_R_4_-R | GAAGATCTCCCAAGTACCTGATATCGTC | 3’deletion analysis |
| P_1_R_5_-R | GAAGATCTCCCAAAGGCAAACTGCAGAT | 3’deletion analysis |
| P1-113-F | GGGGTACCGAGGAACATTCTACTTGG | For induced minimal promoter |
| P1-113-R | GAAGATCTGAACACATGTGGTTAGGT | For induced minimal promoter |
| EF1a-QF | TATGATTACTGGTACCTCCC | RT-PCR(tobacco internal control)) |
| EF1a-QR | ACCTAGCCTTGGAATACTTG | RT-PCR (tobacco internal control)) |
| mGUS-F | ATCCGGTCAGTGGCAGTGAAGG | RT-PCR |
| mGUS-R | CAGCGTAAGGGTAATGCGAG | RT-PCR |
| Gmg1-F | TTTGAGGTTGGGACTAACGG | RT-PCR for *GmaPPO12* gene |
| Gmg1-R | ATCTTGATGCCTCGGATGTT | RT-PCR for *GmaPPO12* gene |
| 2281-F | TGATGAAGATGATAAGTTGGTCA | RT-PCR for *GmaPPO12* homologous gene |
| 2281-R | CAATCTTCTTGTTGTTTTTGTGA | RT-PCR for *GmaPPO12* homologous gene |
| ACT20-F | TCGAGGCACCTAATTCTTGG | RT-PCR(soybean internal control)) |
| ACT20-R | GTGTCTGGATTGGTGGCTCT | RT-PCR(soybean internal control) |

a:The underlined italicised sites are the sites for the digestion of restriction enzymes *Bgl*II

b:The italicised sites are the sites for the digestion of restriction enzymes *Stu*I

c:The bold words are the sites for the digestion of restriction enzymes *Kpn*I
