# Supplementary figures and images for: Identification and Functional Characterization of the Soybean GmaPPO12 Promoter Conferring Phytophthora sojae Induced Expression
Source: PLoS One. 2013 Jun 28;8(6):e67670. doi: 10.1371/journal.pone.0067670 (PMC3695865; doi:10.1371/journal.pone.0067670)

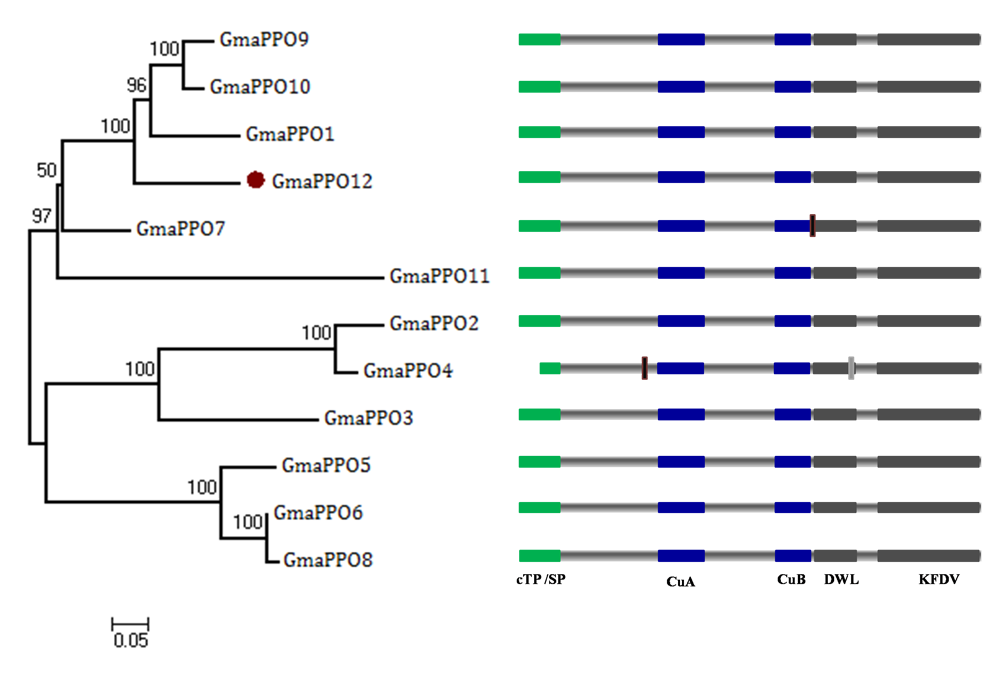

Supplement: Figure S1 — The ClustalW multiple sequence alignment was formed using the deduced soybean PPO proteins. The tree was constructed from the ClustalW alignment using the neighbor-joining method of the MEGA program. The scale bar represents 0.05 substitutions per site and the numbers next to the nodes are bootstrap values from 1,000 replicates. Predicted targeting sequences are colored green (chloroplast transit peptide), black (signal peptide), or gray (unknown). The CuA and CuB domains are colored blue, and C-terminal conserved areas are dark gray. Approximate intron positions are shown as vertical bars, mapped onto the predicted protein. (TIF) [file pone.0067670.s002.tif]

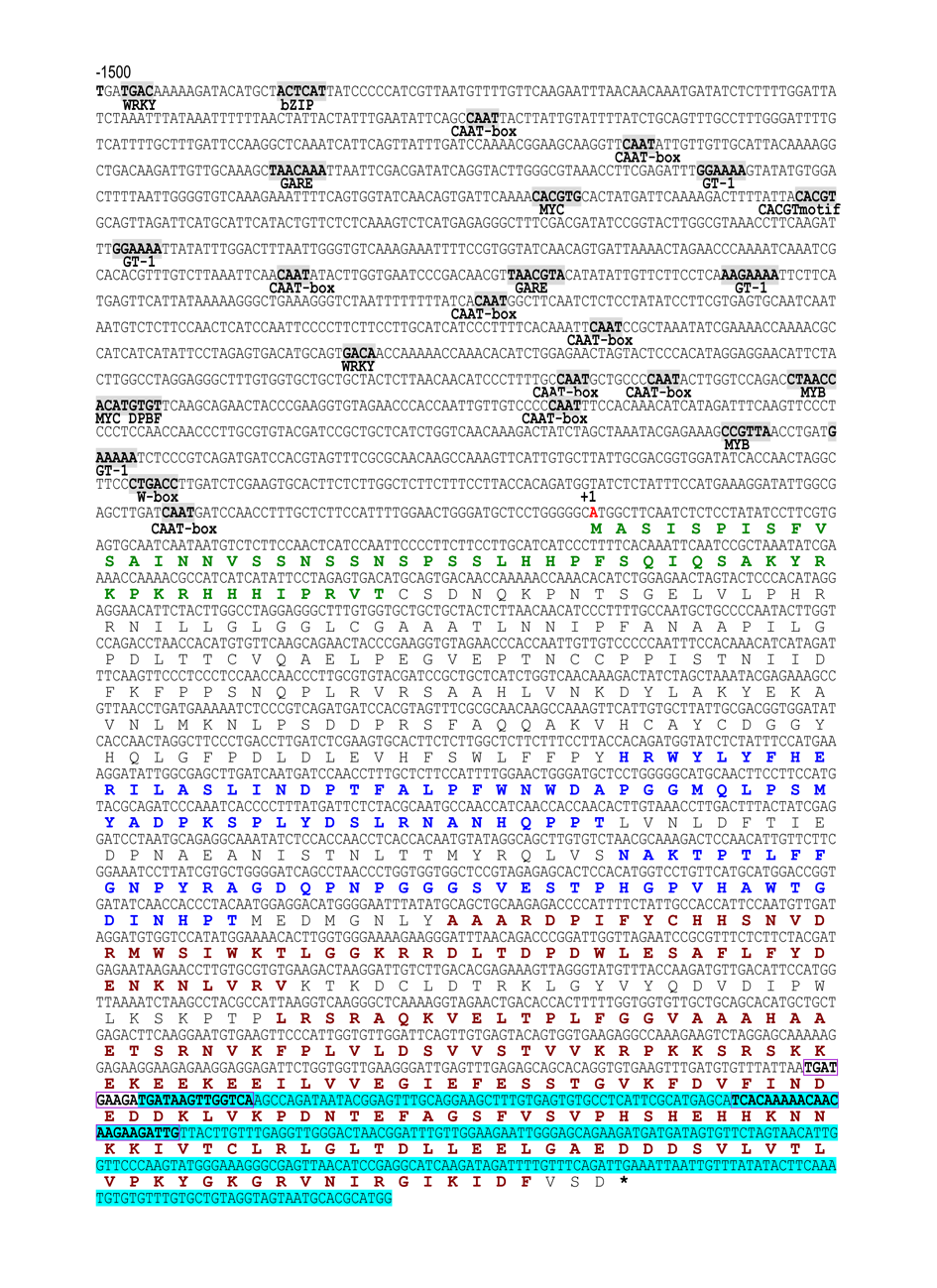

Supplement: Figure S2 — The translational start sites (+1) are shown in red. Upstream of the translation start sites (+1) is the promoter sequence. The promoter motifs with significant similarity to the previously identified cis-acting elements are shaded and the names are given under the elements. The translation initiation codon (M) downstream amino acid sequence contains PPO conserved regions: the N-terminal chloroplast transit peptide sequence is colored green, CuA and CuB domain sequences are colored blue, C-terminal DWL and KFDV domains are colored red, and unknown sequences are colored gray. The nucleotide sequences colored blue-green are microarray EST. The sequence in purple boxes indicates the primers for quantitative RT-PCR to detect the inducibility of GmaPPO12. (TIF) [file pone.0067670.s003.tif]

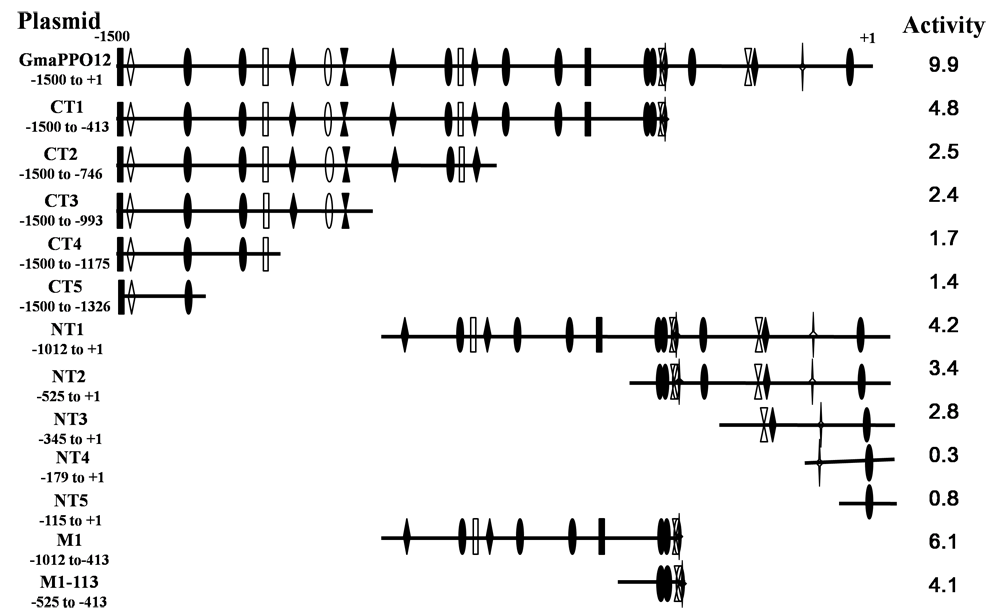

Supplement: Figure S3 — Structural mapping of the deletion mutations containing different cis-elements are presented schematically with a line representing the portion of sequence that was not deleted. The position of the last remaining base of the promoter sequence for each mutant is indicated on the left. The enzymatic activity upon infection of the corresponding deletion mutations is shown at the right. (TIF) [file pone.0067670.s004.tif]
